# Comparative study of efficacy and safety of pulse versus half-pulse steroid therapy for Vogt-Koyanagi-Harada Disease

**DOI:** 10.1007/s10384-025-01213-3

**Published:** 2025-05-26

**Authors:** Kinya Tsubota, Hiroshi Goto, Masaki Asakage, Yuto Kinoshita, Ryota Nonaka, Kei Wakatsuki, Risa Sugawara, Ryosuke Fukai, Yoshihiko Usui

**Affiliations:** Department of Ophthalmology, Tokyo Medical University Hospital, 6-7-1 Nishishinjuku, Shinjuku-ku, Tokyo, 160-0023 Japan

**Keywords:** Vogt-Koyanagi-Harada disease, Uveitis, Corticosteroid therapy, Steroid pulse, Steroid half pulse

## Abstract

**Purpose:**

High-dose steroid therapy is commonly used for Vogt-Koyanagi-Harada (VKH) disease in Japan, but the optimal initial dose remains uncertain. This study investigated the efficacy and safety of conventional steroid pulse therapy (pulse group) and half-dose steroid pulse therapy (half-pulse group) for the initial treatment of VKH disease.

**Study design:**

This was a single-center, retrospective observational study based on a review of medical records.

**Subjects and methods:**

Seventy-two patients with initial-onset VKH disease treated at Tokyo Medical University Hospital were analyzed. The pulse group (44 patients) received 1000 mg/day of intravenous methylprednisolone (MPSL), while the half-pulse group (28 patients) received 500 mg/day of MPSL. In both groups, steroids were tapered according to ocular findings, and the minimum follow-up period was eight months.

**Results:**

Recurrence rate during follow-up period (pulse group: 41%, half-pulse group: 34%, p = 0.48), mean number of recurrences (pulse group: 1.9 ± 1.2, half-pulse group: 1.5 ± 0.9, p = 0.42), and type of recurrence (anterior uveitis: pulse group: 11%, half-pulse group: 17%, p = 0.33; posterior inflammation: pulse group: 34%, half-pulse group: 22%, p = 0.14) were not significantly different between the two groups. The proportion of patients requiring cataract surgery during the follow-up period tended to be higher in the pulse group (pulse group: 10%, half-pulse group: 3%, p = 0.12), although not significantly different. The incidence of sunset glow fundus was not significantly different between the two groups (pulse group: 23%, half-pulse group: 10%, p = 0.22).

**Conclusions:**

No significant difference in clinical efficacy for VKH disease was observed between conventional steroid pulse therapy and half-pulse therapy, and by reducing the total steroid dose, half-pulse therapy potentially lowers the risk of adverse effects related corticosteroid.

**Supplementary Information:**

The online version contains supplementary material available at 10.1007/s10384-025-01213-3.

## Introduction

Vogt-Koyanagi-Harada (VKH) disease is a non-infectious uveitis caused by autoimmune response against melanocyte-containing tissues including the uvea, meninges, inner ear, and skin. The disease was first described by Alfred Vogt in 1906 [1], and was subsequently reported from various countries worldwide including Japan [2–4]. In the acute phase, VKH disease causes various symptoms including bilateral granulomatous anterior uveitis, exudative retinal detachment, and optic disc hyperemia and swelling, leading to sudden visual loss [2].

High-dose corticosteroid therapy is effective in the early phase of VKH disease [5, 6]. Although steroid pulse therapy with 1000 mg of methylprednisolone for 3 days has been widely used in Japan [7], oral prednisolone at doses of 0.5–1 mg/kg/day is used in approximately 80% of the cases in the United States and approximately 90% in France [8, 9]. Thus, the optimal dosage of corticosteroids for early-phase treatment remains a subject of debate.

Conventional steroid pulse therapy used in Japan is effective for early recovery of visual function by rapid suppression of intraocular inflammation in the acute phase. However, there are concerns over the adverse effects related to high-dose corticosteroids. These adverse effects include increased risk of infections, osteoporosis, diabetes, hypertension, and psychiatric symptoms, which potentially have important impact on patients’ quality of life [10, 11]. As a result, alternative regimens using lower doses of corticosteroids, such as mini-dose pulse therapy (100 mg of methylprednisolone) or half-dose pulse therapy (500 mg of methylprednisolone), have gained attention [12, 13]. These therapies aim to reduce the adverse effects caused by high-dose pulse therapy while maintaining equivalent therapeutic efficacy. Half-dose pulse therapy is expected to reduce the risk of long-term adverse effects by lowering the total dose of corticosteroids compared to conventional pulse therapy [12, 13]. However, few studies have compared the efficacy and safety of half-dose pulse therapy with those of conventional pulse therapy. If half-dose pulse therapy can achieve comparable efficacy with a much lower steroid burden compared to conventional pulse therapy, the clinical significance would be substantial.

This study aimed to establish an optimal treatment regimen for VKH disease by comparing the clinical efficacy and safety of conventional steroid pulse therapy with half-dose pulse therapy.

## Methods

We conducted a retrospective, single-institution, observational study. This study was conducted in compliance with the Helsinki principles. Ethical approval was obtained from the Medical Ethical Research Committee of Tokyo Medical University Hospital (Approval No.: T2021-0001). Written informed consent was obtained from all the subjects of this study.

This study included 146 eyes of 73 patients diagnosed with VKH disease between May 2018 and May 2022 at Tokyo Medical University Hospital, who underwent initial treatment and were followed for at least 8 months. VKH disease was diagnosed according to the Revised Diagnostic Criteria for Vogt-Koyanagi-Harada Disease [14]. Patients were divided into two groups: those who received conventional steroid pulse therapy with 1000 mg/day of methylprednisolone (MPSL) for 3 days as initial treatment (pulse group), and those who received half-dose corticosteroid pulse therapy with 500 mg/day of MPSL for 3 days as initial treatment (half-pulse group). The clinical course after treatment was evaluated retrospectively. Between May 2018 and December 2020, all patients who were diagnosed with VKH disease for the first time were in principle treated with conventional corticosteroid pulse therapy. From January 2021 onward, the treatment was changed to half-dose corticosteroid pulse therapy, except in patients weighing 80 kg or more, who were treated with conventional corticosteroid pulse therapy and were excluded from the present study. After 3 days of intravenous infusion, treatment was switched to oral prednisolone in both treatment groups. The prednisolone dose was tapered gradually according to symptoms and ocular findings.

The following clinical data were collected from medical records: age, sex, body weight, ocular findings, systemic findings, best-corrected visual acuity (BCVA) at initial presentation, presence or absence and frequency of recurrence of intraocular inflammation, type of recurrence, cataract surgery conducted during the follow-up period, adverse events by treatment, speed of oral prednisolone tapering, duration of steroid therapy until discontinuation, and total cumulative steroid dose calculated as prednisolone equivalent. The above clinical variables were analyzed statistically.

To evaluate the effect of the difference in pulse therapy on recurrence, a logistic regression analysis was performed using recurrence as the dependent variable. The independent variables included the initial pulse therapy regimen, age, sex, body weight, initial BCVA, speed of oral prednisolone tapering, and duration of steroid therapy until discontinuation. Recurrence of intraocular inflammation was defined as the presence of anterior chamber inflammation, retinal pigment epithelial folds observed by optical coherence tomography (OCT), exudative retinal detachment, and optic disc hyperemia and swelling during the course of disease.

## Treatment protocol

In the pulse group, patients first received 1000 mg/day of intravenous MPSL for three days, followed by oral prednisolone (1 mg/kg/day, up to a maximum dose of 60 mg/day). In the half-pulse group, patients received 500 mg/day of intravenous MPSL for 3 days, followed by oral prednisolone (1 mg/kg/day, up to a maximum of 60 mg/day). After initiating oral prednisolone, the dosage was tapered by approximately 5 mg every two weeks. Once the dose reached 20 mg/day or lower, the tapering speed was adjusted according to symptoms and ocular findings, and the treatment was discontinued after a minimum of 6 months. When anterior chamber inflammation was clearly observed at initial presentation, betamethasone 0.1% eye drops and tropicamide were added; these were discontinued after inflammation subsided. If exudative retinal detachment remained after pulse or half-pulse therapy, betamethasone (6–8 mg/day) intravenous infusion was administered before switching to oral prednisolone. During follow-up, when intraocular inflammation recurred, treatment was adjusted according to symptoms and ocular findings, including the initiation or increased frequency of betamethasone 0.1% eye drops, posterior sub-Tenon injection of triamcinolone acetonide, and dose increase of oral prednisolone. For patients experiencing two or more recurrences, concurrently with the dose increase of oral prednisolone, adjunctive therapy with oral methotrexate (8 mg/week) or oral cyclosporine (3–5 mg/kg/day) was initiated.

## Statistical analysis

Comparison between the two groups was performed using Welch's t-test or Fisher's exact test. Factors associated with recurrence were analyzed using binary logistic regression analysis. A *p* value less than 0.05 was considered statistically significant. All analyses were performed using commercial statistical analysis software (BellCurve for Excel, Social Survey Research Information Co. Ltd.; JMP, SAS Institute, Ltd.).

## Results

Table 1 shows a comparison of patient background and visual outcome between the two treatment groups. The pulse group consisted of 44 patients (88 eyes), while the half-pulse group had 29 patients (58 eyes). The male-to-female ratio was 1:1 in the pulse group and 1:2.2 in the half-pulse group, with a significant difference between the two groups (p = 0.02). There were no significant differences between the two groups in terms of mean age, mean body weight, ocular findings, systemic findings, and initial BCVA at the start of treatment. However, the mean follow-up period was significantly longer in the pulse group (p < 0.01). In this study, one patient in the pulse group underwent a second course of steroid pulse therapy. Visual outcomes did not differ significantly between the two groups at all time points: 1 week, 1 month, and 6 months after treatment initiation, and at the final visit. The case requiring the second pulse therapy is presented in Supplemental Fig. 1. Furthermore, the half-pulse group did not require a longer period of time to achieve visual improvement compared to the pulse group.

**Table 1:** Patient background and visual outcome

	Pulse group	Half-pulse group	*p* value
Number of patients, *n*	44	29	
Sex: male/female, *n*	22/22	9/20	0.02
Age, years	42	45	0.20
Body weight, kg	62.9	59.9	0.21
Follow-up period, months	22.3	14.5	<0.01
Ocular findings at first visit			
Anterior chamber cells, grade	0.77 ± 0.75	1.03 ± 0.76	0.83
Optic disc hyperemia and swelling, n (%)	65 (74%)	34 (59%)	0.07
Exudative retinal detachment, *n* (%)	81 (92%)	52 (90%)	0.77
Retinal pigment epithelial folds, *n* (%)	78 (89%)	54 (93%)	0.56
Systemic findings at first visit			
Headache or meningismus, *n* (%)	28 (63%)	24 (86%)	0.05
Tinnitus or dysacusis, *n* (%)	8 (18%)	6 (21%)	0.50
Vitiligo, *n* (%)	0 (0%)	0 (0%)	
Poliosis, *n* (%)	0 (0%)	0 (0%)	
BCVA (logMAR)			
first visit	0.23 ± 0.35	0.17 ± 0.37	0.35
1 week after starting	0.08 ± 0.21	0.09 ± 0.26	0.65
1 month after starting	− 0.02 ± 0.14	− 0.03 ± 0.18	0.84
6 months after starting	− 0.06 ± 0.17	− 0.08 ± 0.08	0.35
final visit	− 0.09 ± 0.13	− 0.09 ± 0.05	0.96

*BCVA* best corrected visual acuity

Table 2 compares the rates of recurrence of intraocular inflammation and complications between the two groups. The rate of recurrence of intraocular inflammation was 41% in the pulse group and 34% in the half-pulse group (p = 0.48), and the mean number of recurrences was 1.9 in the pulse group and 1.5 in the half-pulse group (p = 0.42). Regarding the type of recurrence, anterior uveitis alone was observed in 11% of the eyes in the pulse group and 17% in the half-pulse group (p = 0.33), while posterior inflammation was found in 34% in the pulse group and 22% in the half-pulse group (p = 0.14). The incidence of sunset glow fundus was 23% in the pulse group and 10% in the half-pulse group (p = 0.22), with no significant difference between the two groups. The frequency of cataract surgery performed during the follow-up period did not differ significantly between the two groups, although there was a tendency of lower frequency in the half-pulse group (2 eyes, 3%) compared to the pulse group (9 eyes, 10%) (p = 0.12).

**Table 2:** Recurrence of intraocular inflammation and complications

	Pulse group	Half-pulse group	*p* value
Recurrence, *n* (%)	36 eyes (41%)	20 eyes (34%)	0.48
Mean number of recurrences	1.9	1.5	0.42
Type of recurrence			
Anterior inflammation, *n* (%) *	10 eyes (11%)	10 eyes (17%)	0.33
Posterior inflammation, *n* (%) *	30 eyes (34%)	13 eyes (22%)	0.14
Complications			
Cataract surgery, n (%)	9 eyes (10%)	2 eyes (3%)	0.12
Sunset glow fundus, n (%)	20 eyes (23%)	6 eyes (10%)	0.22

*With overlap

Systemic adverse effects in the two groups are summarized in Table 3. No significant differences were observed between the two groups in terms of adverse effects occurring within one month after treatment initiation and throughout the follow-up period. There were also no significant differences in laboratory abnormalities including HbA1c levels after initial treatment, and abnormal blood test results during the course of follow-up. The most frequently observed systemic adverse effect was hepatic dysfunction due to elevated alanine aminotransferase levels. However, in all cases, the adverse effects improved by reducing the corticosteroid dose alone.

Table 4 presents a comparison of the treatment contents between the two groups. In this study, all patients were able to discontinue corticosteroid therapy. The mean duration until discontinuation of oral prednisolone was 8.7 months in the pulse group and 8.0 months in the half-pulse group, with no significant difference between the two groups (p = 0.22). Additionally, there was no significant difference between the two groups in the rate of initiation of methotrexate (p = 0.64) or cyclosporine (p = 0.68) for recurrence of intraocular inflammation. The total cumulative corticosteroid dose was consistently lower in the half-pulse group compared to the pulse group for all the durations from the start of treatment (p < 0.01).

**Table 3:** Recurrence of intraocular inflammation and complications

	Pulse group	Half-pulse group	*p* value
Adverse events of treatment within the first month, n (%)	17 (38%)	14 (34%)	0.47
Type of adverse events within the first month			
Abnormality liver enzyme, n (%) *	4 (9%)	4 (14%)	
Sleeplessness, n (%) *	3 (7%)	0 (0%)	
Muscle cramp, n (%) *	3 (7%)	0 (0%)	
Hyperglycemia, n (%) *	1 (2%)	2 (7%)	
Hyperlipidemia, n (%) *	1 (2%)	3 (10%)	
Others, n (%) *	5 (11%)	6 (21%)	
Adverse events of treatment in follow up period, n (%)	30 (68%)	18 (62%)	0.62
Type of adverse events in follow up period			
Abnormality liver enzyme, n (%) *	8 (18%)	6 (21%)	
Sleeplessness, n (%) *	4 (9%)	1 (3%)	
Muscle cramp, n (%) *	5 (11%)	4 (14%)	
Hyperglycemia, n (%) *	8 (18%)	7 (24%)	
Hyperlipidemia, n (%) *	6 (14%)	4 (14%)	
Others, n (%) *	13 (30%)	8 (28%)	
HbA1c after initial treatment, %	6.0 ± 1.0	6.1 ± 0.9	0.50
Abnormal in peripheral blood examination in follow up period, n (%)	18 (41%)	13 (45%)	0.81

*With overlap

**Table 4:** Treatments

	Pulse group	Half-pulse group	*p* value
Duration until discontinuation of oral prednisolone, months	8.7	8.0	0.22
Cyclosporine initiation, *n* (%)	3 cases (7%)	3 cases (10%)	0.68
Methotrexate initiation, *n* (%)	4 cases (9%)	1 case (2%)	0.64
Biological (adalimumab) initiation, *n* (%)	3 cases (7%)	3 cases (10%)	0.68
Mean total dose of systemic steroids			
first 1 week of treatment, mg	3790	2033	< 0.01
first 2 weeks of treatment, mg	5304	3033	< 0.01
first 4 weeks of treatment, mg	5922	3519	< 0.01
from start to end of treatment, mg	9162	5776	< 0.01

To evaluate the possible effect of difference in initial steroid pulse therapy regimen (pulse vs. half-pulse) on the recurrence of intraocular inflammation, a binary logistic regression analysis was conducted in all cases with recurrence as the dependent variable (Table 5). The analysis identified age (p = 0.03) and body weight (p = 0.04) as significant risk factors for recurrence, indicating that older patients and patients with higher body weight were more likely to experience recurrence. In contrast, difference in pulse therapy regimen (pulse vs. half-pulse) was not associated with the risk of recurrence (p = 0.48). Additionally, the duration of prednisolone treatment until discontinuation was found to be a factor associated with recurrence (p < 0.01). An analysis of all patients comparing the duration of prednisolone treatment between patients with and those without recurrence showed that the mean duration tended to be longer in those with recurrence (9.1 months) compared to those without recurrence (8.4 months), although the difference was not statistically significant (p = 0.18). At the time of recurrence, the mean dose of prednisolone was 3.5 ± 7.5 mg.

**Table 5:** Clinical factors contributing to recurrence (all patients, n = 72)

	*p* value	Odds ratio	95% CI
Sex	0.07	1.06	− 2.01 ~ 0.10
Age	< 0.01	1.03	0.05 ~ 0.06
Weight	< 0.01	1.07	0.03 ~ 0.12
BCVA at 1st visit	0.18	0.48	− 1.82 ~ 0.36
Pulse therapy regimen (pulse vs. half-pulse)	0.48	0.03	− 12.5 ~ 5.9
Total corticosteroid dose (1 week)	0.23	1.00	− 0.02 ~ 0.08
Total corticosteroid dose (2 weeks)	0.36	1.00	− 0.04 ~ 0.01
Total corticosteroid dose (4 weeks)	0.73	1.00	− 0.02 ~ 0.01
Duration until the end of systemic steroid medication	< 0.01	1.40	0.12 ~ 0.55
BCVA best corrected visual acuity.			

Next, we redefined the time of recurrence as that when prednisolone treatment was discontinued and performed another binary logistic regression analysis with recurrence as the dependent variable (Table 6). The results showed that the duration (days) until discontinuation of oral prednisolone was not a risk factor of recurrence (p = 0.85). However, the odds ratio for the duration (days) taken to taper prednisolone to 5 mg was 0.96, with a *p* value less than 0.01. In other words, the longer the duration required to taper prednisolone to 5 mg, the lower was the risk of recurrence.

**Table 6:** Dose and duration of prednisolone use contributing to recurrence

	*p* value	Odds ratio	95% CI
Duration to taper dose to 40 mg prednisolone	0.89	1.00	− 0.05 ~ 0.04
Duration to taper dose to 30 mg prednisolone	0.48	1.00	− 0.10 ~ 0.05
Duration to taper dose to 20 mg prednisolone	0.49	1.02	− 0.03 ~ 0.07
Duration to taper dose to 10 mg prednisolone	0.28	1.02	− 0.01 ~ 0.05
Duration to taper dose to 5 mg prednisolone	< 0.01	0.96	− 0.05 ~ -0.01
Duration until the end of systemic steroid medication	0.85	0.99	− 0.01 ~ -0.01

To further examine the specific number of days required to taper prednisolone to 5 mg/day, we generated a receiver operating characteristic curve using the duration to taper prednisolone to 5 mg as the predictor, and recurrence as the outcome variable including all cases in the analysis (Supplemental Fig. S2). The area under the curve (AUC) was 0.64 and the cutoff value was 154 days, indicating that maintaining a prednisolone dose of 5 mg/day or higher for 154 days or more significantly reduced the risk of recurrence. We next compared the recurrence rates between the group that maintained prednisolone at 5 mg/day or higher for 154 days or more and the group that maintained it for less than 154 days; the former group had a significantly lower recurrence rate (p < 0.01) (Supplemental Table S2). Furthermore, although both groups had generally good visual outcome following treatment, the group that maintained prednisolone at 5 mg/day or higher for 154 days or more had significantly better visual outcome at 6 months after starting treatment (p = 0.01) and at the final follow-up visit (p < 0.01).

In summary, half-dose steroid pulse therapy for VKH disease was found to achieve treatment outcomes comparable to conventional steroid pulse therapy. By reducing the total steroid dose using half-dose pulse therapy, mitigation of the risk of steroid-related adverse effects may be expected. Additionally, analyses showed that whether the pulse therapy was conventional or half-dose (pulse vs. half-pulse) was not a contributor to recurrence, while maintaining a prednisolone dose of 5 mg/day or higher for at least 154 days reduced the risk of recurrence.

## Discussion

When VKH disease was first reported in 1906, treatment was primarily symptomatic [1–4]. Three decades later, cortisone was extracted from the adrenal glands by Kendall, and the chemical structure of cortisone was elucidated in the 1940s by Reichstein, while Hench was the first to apply cortisone as a therapeutic agent in patients with rheumatoid arthritis [15, 16]. This breakthrough highlighted the potent anti-inflammatory effects of cortisone [16]. In 1954, Cordes administered cortisone for the first time to a Japanese patient with VKH disease, and the effectiveness of cortisone in treating VKH disease was confirmed [17].

In 1969, Masuda and Tanishima [5] reported the efficacy of initial treatment with 200 mg of prednisolone, although a certain percentage of cases experienced recurrence [18]. In the 1980s, the efficacy of combination therapy with corticosteroids and cyclosporine was reported [19, 20]. However, the time required for cyclosporine to become effective and the occurrence of adverse effects such as renal dysfunction presented challenges. Subsequently, steroid pulse therapy, developed in 1969 to suppress rejection in recipients of kidney transplantation [21], was applied to VKH disease and became established as a standard treatment for VKH disease in Japan [6, 7]. In contrast, in Western countries oral corticosteroid therapy is primarily used for VKH disease [8, 9]. Studies comparing oral corticosteroids (1 mg/kg) with steroid pulse therapy report fewer recurrences in the steroid pulse therapy group [22]. However, the adverse effects associated with high-dose steroid therapy remain a concern [10, 11]. Consequently, half-dose and mini-dose steroid pulse therapy that aimed at reducing the total steroid dose have gained attention in the treatment of other inflammatory diseases [12, 13]. However, no previous studies report the usefulness of half-pulse therapy for VKH disease, which prompted us to conduct the present study with the objective of comparing the clinical efficacy and adverse effects of half-pulse therapy with conventional pulse therapy.

Our analyses showed no significant differences between conventional pulse therapy and half-pulse therapy in terms of recurrence rate, visual outcome, incidence of sunset glow fundus, and the occurrence of adverse effects in VKH disease. Furthermore, based on self-reported outcomes by patients, both treatment groups demonstrated a similar trend of early improvement in headache and hearing loss, typically within 1–2 days after treatment initiation. These results suggest that the therapeutic effects on systemic symptoms were comparable between the two treatment groups. In other words, both treatment methods demonstrated equal efficacy in managing the acute and chronic phases of VKH disease. Notably, visual improvement was comparable in the two groups at all the time points during the course of follow-up, indicating that intraocular inflammation can be controlled effectively by either of the two treatments. Meanwhile, cases requiring additional treatment due to recurrence shortly after initial steroid pulse therapy have been reported [23]. Similarly, in our study, one case in the conventional steroid pulse therapy group required a second course of pulse therapy. This finding suggests that even today, high-dose corticosteroid therapy plays an important role in the treatment of VKH disease. In this study, although the incidence of adverse effects was apparently slightly higher in the conventional pulse therapy group compared to the half-pulse therapy group, the difference was not statistically significant. Half-pulse therapy, however, significantly reduces the total steroid dose, suggesting the possibility that the risk of adverse effects may be mitigated. Given that both treatments have equivalent clinical efficacy, it is preferable to use the treatment with fewer adverse effects. Fortunately, no serious systemic adverse events were observed in either group in the present study, but the potential to reduce the risk of complications such as infections, osteoporosis, hypertension, and diabetes is an important advantage for patients requiring long-term treatment. There was a tendency for a lower rate of cataract surgery in the half-pulse group compared to the pulse group (3% vs. 10%, p = 0.12), although further verification is needed due to the significant difference in follow-up period between the two treatment groups.

In this study, age and body weight were identified as significant risk factors for the recurrence of VKH disease. Specifically, older patients (p = 0.03) and patients with higher body weight (p = 0.04) were more likely to experience recurrence. While men generally have higher body weight than women, sex was not identified as a significant risk factor for recurrence (p = 0.07). On the other hand, the duration of prednisolone therapy was found to be associated with suppression of recurrence. The longer the period taken to taper prednisolone to a dose of 5 mg/day or less, the lower was the risk of recurrence (p < 0.01). Given that this study adopted a protocol in which prednisolone was tapered by 5 mg every 2 weeks, and that the average dose of prednisolone at the time of recurrence was 3.5 ± 7.5 mg, it is suggested that maintaining a dose of 10 mg/day or more for a longer duration may help prevent recurrence. These findings imply that for patients at a higher risk of recurrence, adjusting the tapering speed of oral prednisolone may be an effective strategy to prevent relapse.

This study has several limitations. First, as a retrospective observational study, it is more susceptible to bias compared to prospective randomized controlled trials. Additionally, the relatively small number of patients may have limited the statistical power of the findings. Patients weighing over 80 kg were excluded from the half-pulse group. Hence, the efficacy of this treatment in overweight patients remains unknown. One case of COVID-19 vaccination- or infection-related VKH disease has been reported after 2020 [24]. In the present study, we obtained the COVID-19 infection or vaccination history from all patients diagnosed with VKH disease after 2020, and no cases of VKH disease associated with COVID-19 vaccination or COVID-19 infection were included. However, since vaccination history was self-reported by patients, we cannot completely exclude the possibility that the cohort contained some cases of vaccine-induced VKH disease. The difference in treatment response between vaccine-induced VKH disease and conventional VKH disease remains unclear, and the extent to which COVID-19 may have influenced the outcomes in this study is uncertain. In addition, the efficacy and safety of half-dose corticosteroid pulse therapy in patients with COVID-19 vaccination or infection-related VKH disease remain unknown. Moreover, since this study was conducted at a single institution, further research is needed to confirm the generalizability of the present results to other centers. Future studies should include prospective randomized controlled trials with a larger sample to further evaluate the efficacy and safety of half-pulse therapy. In particular, it will be important to establish treatment regimens taking into account risk factors of recurrence, as well as to explore optimal use of immunosuppressive agents. Furthermore, long-term evaluations of adverse effects and the impact on patients' quality of life should be examined.

By comparing conventional steroid pulse therapy with half-pulse therapy for VKH disease, this study demonstrates that the two therapies have equivalent clinical efficacy, and half-pulse therapy potentially lowers the risk of steroid-related adverse effects through reducing the total steroid dose.

## Supplementary Information

Below is the link to the electronic supplementary material.

Supplementary file1 (PPTX 1118 KB)

Supplementary file2 (DOCX 16 KB)

## References

[1] Vogt A. Frühzeitiges ergrauen der Zilien und Bemerkungen über den sogenannten plötzlichen Eintritt dieser Veränderung. Klin Monatsbl Augenheilkd. 1906;44:228–42 (**(in German)**).

[2] Herbort CP, Mochizuki M. Vogt-Koyanagi-Harada disease: inquiry into the genesis of a disease name in the historical context of Switzerland and Japan. Int Ophthalmol. 2007;27:67–79. 17468832 10.1007/s10792-007-9083-4

[3] Komoto J. Ueber Vitiligo und Auge. Klin Monatsbl Augenheilkd. 1911;49:139–42 (**(in German)**).

[4] Koyanagi Y. Budoumakuen ni tomonau mouhatsu no datsuraku hakuutsu ni tsuite. Nippon Ganka Gakkai Zasshi. 1914;18:1188 (**(in Japanese)**).

[5] Masuda K, Tanishima T. Early treatment of Harada’s disease. Rinsho Ganka. 1969;23:553 (**(in Japanese)**).

[6] Kotake S, Ohno S. Pulse methyl prednisolone therapy in the treatment of Vogt-Koyanagi-Harada disease. Rinsho Ganka. 1984;38:1053–8 (**(in Japanese)**).

[7] Sasamoto Y, Ohno S, Matsuda H. Studies on corticosteroid therapy in Vogt-Koyanagi-Harada disease. Ophthalmologica. 1990;201:162–7. 2089358 10.1159/000310145

[8] Reddy AK, John FT, Justin GA, Dahr SS. Vogt-Koyanagi-Harada disease in a native American population in Oklahoma. Int Ophthalmol. 2021;41:915–22. 33403519 10.1007/s10792-020-01647-3

[9] Diallo K, Revuz S, Clavel-Refregiers G, Sené T, Titah C, Gerfaud-Valentin M, et al. Vogt-Koyanagi-Harada disease: a retrospective and multicentric study of 41 patients. BMC Ophthalmol. 2020;20:395. 33028239 10.1186/s12886-020-01656-x PMC7539440

[10] Schacke H, Docke WD, Asadullah K. Mechanisms involved in the side effects of glucocorticoids. Pharmacol Ther. 2002;96:23–43. 12441176 10.1016/s0163-7258(02)00297-8

[11] Hox V, Lourijsen E, Jordens A, Aasbjerg K, Agache I, Alobid I, et al. Benefits and harm of systemic steroids for short- and long-term use in rhinitis and rhinosinusitis: an EAACI position paper. Clin Transl Allergy. 2020;10:1. 31908763 10.1186/s13601-019-0303-6 PMC6941282

[12] Iglehart IW 3rd, Sutton JD, Bender JC, Shaw RA, Ziminski CM, Holt PA, et al. Intravenous pulsed steroids in rheumatoid arthritis: a comparative dose study. J Rheumatol. 1990;17:159–62. 2319516

[13] Franchin G, Diamond B. Pulse steroids: how much is enough? Autoimmun Rev. 2006;5:111–3. 16431338 10.1016/j.autrev.2005.08.003

[14] Read RW, Holland GN, Rao NA, Tabbara KF, Ohno S, Arellanes-Garcia L, et al. Revised diagnostic criteria for Vogt-Koyanagi-Harada disease: report of an international committee on nomenclature. Am J Ophthalmol. 2001;131:647–52. 11336942 10.1016/s0002-9394(01)00925-4

[15] Harold L, Charles SM, Edward CK. The chemistry of crystalline substances isolated from the suprarenal gland. J Biol Chem. 1936;114:613–31.

[16] The RTN, Nobel chronicles. Edward Calvin Kendall (1886–1972); Philip Showalter Hench (1896–1965); and Tadeus Reichstein (1897–1996). Lancet. 1950;1999(353):1370. 10.1016/s0140-6736(05)74374-9 10218568

[17] Cordes FC. Harada’s disease treated with cortisone: report of a typical case. Trans Am Ophthalmol Soc. 1954;52:79–104. 13274418 PMC1312587

[18] Hayasaka S, Okabe H, Takahashi J. Systemic corticosteroid treatment in Vogt-Koyanagi-Harada disease. Graefes Arch Clin Exp Ophthalmol. 1982;218:9–13. 7056483 10.1007/BF02134092

[19] Harada T, Sugita K, Saito A, et al. Traitement des uvéites sévères par la ciclosporine A. Ophthalmologica. 1987;195:21–5 (**(in French)**). 3658332 10.1159/000309775

[20] Wakatsuki Y, Kogure M, Takahashi Y, Oguro Y. Combination therapy with cyclosporin A and steroid in severe case of Vogt-Koyanagi-Harada’s disease. Jpn J Ophthalmol. 1988;32:358–60. 3230722

[21] Kountz SL, Cohn R. Initial treatment of renal allografts with large intrarenal doses of immunosuppressive drugs. Lancet. 1969;1:338–40. 4179352 10.1016/s0140-6736(69)91299-9

[22] Accorinti M, Saturno MC, Iannetti L, Manni P, Mastromarino D, Pirraglia MP. Treatment and prognosis of Vogt-Koyanagi-Harada disease: real-life experience in long-term follow-up. J Clin Med. 2022;11:3632. 35806916 10.3390/jcm11133632 PMC9267436

[23] Muto T, Sakamoto M, Machida S, Imaizumi S. Vogt-Koyanagi-Harada disease recurred after 8 days of initial steroid pulse therapy: a case report. Clin Case Rep Int. 2023;7:1543.

[24] Dutta Majumder P, Sadhu S, González-López JJ, Mochizuki M. A COVID-19 perspective of Vogt-Koyanagi-Harada disease. Indian J Ophthalmol. 2023;71:2587–91. 37322685 10.4103/IJO.IJO_172_23 PMC10417979

