# Supplementary figures and images for: Comparative study of efficacy and safety of pulse versus half-pulse steroid therapy for Vogt-Koyanagi-Harada Disease
Source: Jpn J Ophthalmol. 2025 May 26;69(5):805–12. doi: 10.1007/s10384-025-01213-3 (PMC12391172; doi:10.1007/s10384-025-01213-3)

## Slide 1
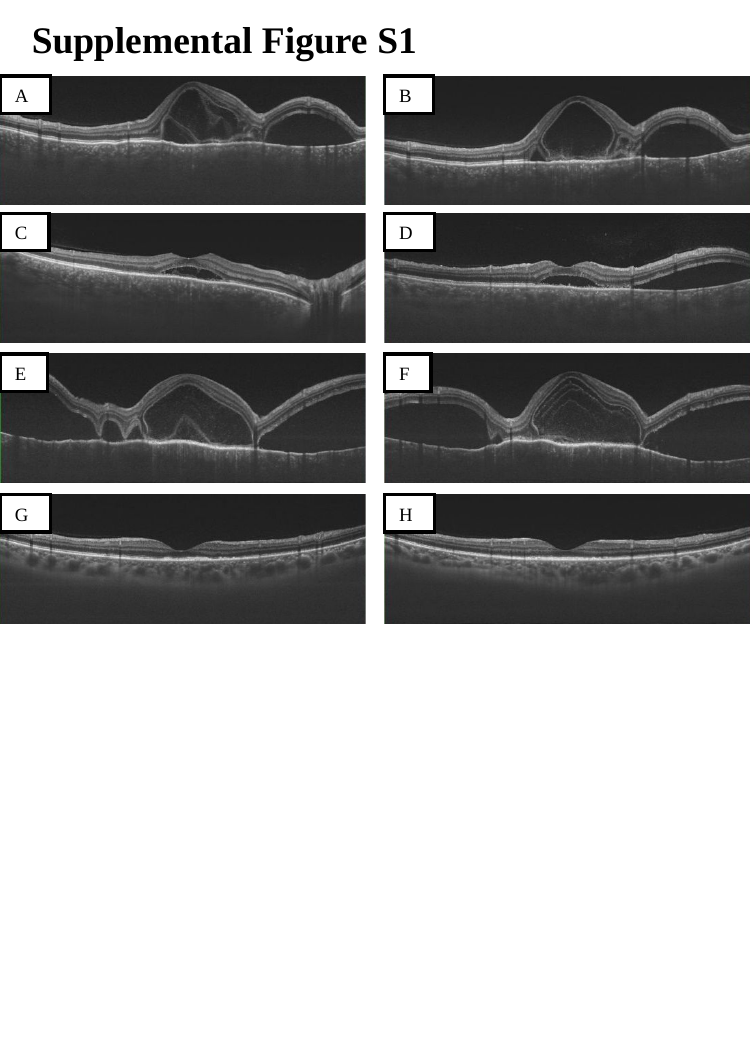

Supplemental Figure S1
A
B
C
D
E
F
G
H

Supplement: Supplementary file 1 — Supplementary file1 (PPTX 1118 KB) [file 10384_2025_1213_MOESM1_ESM.pptx]
