# Supplementary material for: Comparative study of efficacy and safety of pulse versus half-pulse steroid therapy for Vogt-Koyanagi-Harada Disease
Source: Jpn J Ophthalmol. 2025 May 26;69(5):805–12. doi: 10.1007/s10384-025-01213-3 (PMC12391172; doi:10.1007/s10384-025-01213-3)
Supplement: Supplementary file 2 — Supplementary file2 (DOCX 16 KB) [file 10384_2025_1213_MOESM2_ESM.docx]

| Supplemental Table S1. Comparison of recurrence rate between maintaining prednisolone 5 mg or higher for ≥154 days or more and for < 154 days | | | |
| --- | --- | --- | --- |
|  | ≥154 days | < 154 days | *p* value |
| Number of patients, n | 37 | 36 |  |
| Sex: male/female, n | 14/23 | 17/19 | 0.48 |
| Age, years | 44 | 43 | 0.56 |
| Body weight, kg | 60.2 | 63.1 | 0.21 |
| Follow-up period, months | 17.9 | 20.5 | 0.09 |
| Recurrence rate, no. of eyes (%) | 20 (28%) | 36 (50%) | <0.01 |
| BCVA (logMAR) |  |  |  |
| first visit | 0.17 | 0.26 | 0.15 |
| 1 week after starting | 0.08 | 0.10 | 0.67 |
| 1 month after starting | -0.04 | -0.03 | 0.53 |
| 6 months after starting | -0.09 | -0.04 | 0.01 |
| final visit | -0.12 | -0.07 | <0.01 |
| BCVA best corrected visual acuity. |  |  |  |
